# The coiled-coil domain of oncogene RASSF 7 inhibits hippo signaling and promotes non-small cell lung cancer

**DOI:** 10.18632/oncotarget.20223

**Published:** 2017-08-12

**Authors:** Xiaoying Zheng, Qianze Dong, Xiupeng Zhang, Qiang Han, Xu Han, Yong Han, Jingjing Wu, Xuezhu Rong, Enhua Wang

**Affiliations:** ^1^ Department of Pathology, College of Basic Medical Science and First Affiliated Hospital, China Medical University, Shenyang, China; ^2^ Department of Electron Microscopy, Basic Medical College, Chengde Medical College, Chengde, China

**Keywords:** RASSF7, hippo signaling pathway, non-small cell lung cancer, proliferation, migration

## Abstract

Lung cancer is the leading cause of cancer-related deaths worldwide, and despite recent improvements in treatment patient prognosis remains dismal. In this study, we examined the role of N-terminal Ras-association domain family 7 (RASSF7) in human non-small cell lung cancer (NSCLC). We found that RASSF7 was overexpressed NSCLC tissues, which correlated with advanced TNM stage, positive lymph node metastasis, and poor prognosis. This RASSF7 overexpression promoted lung cancer cell proliferation, migration, and invasion. We also found that RASSF7 interacted with mammalian Ste20-like kinase 1(MST1) through its C-terminal coiled-coil domain to inhibit MST1 phosphorylation as well as the phosphorylation of large tumor suppressor kinase 1(LATS1) and yes-associated protein (YAP), while promoting the nuclear translocation of YAP. In addition, RASSF7 overexpression inhibited the Hippo signaling pathway both in *vitro* and *vivo* and promoted the expression of proteins associated with proliferation and invasion, such as connective tissue growth factor. These results suggest that targeting RASSF7 could be exploited for therapeutic benefit in the treatment of NSCLC.

## INTRODUCTION

Lung cancer is the leading cause of tumor-related mortality worldwide [1–3], accounting for approximately one-quarter of the total cancer deaths [4]. Despite recent improvements in lung cancer treatment, the long-term survival rate of patients remains low due to the complexity of the disease, which includes genetic, epigenetic, and environmental factors [5–7]. Identifying new molecular markers and potential therapeutic targets is important for developing more effective treatment strategies that can improve patient outcome.

The Hippo signaling pathway has been proposed to promote tumor development [8, 9]. This pathway is composed of MST1/2, salvador(Sav)1 and Mob1, LATS1/2, YAP/transcriptional coactivator with a PDZ-binding domain (TAZ), and a TEA domain family member (TEAD) [10]. Upstream kinases in this pathway phosphorylate YAP/TAZ, which associates with 14-3-3 protein in the cytoplasm as is thus targeted for ubiquitin-mediated proteasomal degradation. Non-phosphorylated YAP can enter the nucleus and combine with TEAD to activate the transcription of cell proliferation and anti-apoptosis genes.

Ras-association domain family (RASSF) protein interacts with the Hippo pathway [11, 12]. Classical RASSFs (RASSF1–6) have a C-terminal Ras domain (RA) and a Sav/RASSF/Hippo (SARAH) domain. In non-classical RASSFs (RASSF7–10), the RA domain is located at the N terminus [13], and there is no SARAH domain [14]. It was recently predicted that RASSF7, RASSF8, and RASSF10 have a C-terminal coiled-coil (CC) domain that may have a function similar to the SARAH domain [13].

RASSFs share similar functions [12, 15, 16, 17]; these may depend on whether the RA domain is at the C or N terminus or on the presence of a SARAH domain. The pro-apoptotic function of RASSFs is attributed to interactions with MST1/2 mediated by the SARAH domain. However, the extent to which the functions of N-RASSFs depend on specific domains is unclear. The *RASSF7* gene is located on chromosome 11p15 and is expressed in a variety of tissues [18, 19, 20]. RASSF7 has been shown to regulate microtubules and to be necessary for mitotic spindle formation, aurora B kinase activation, and chromosome condensation [19]. It can also inhibit the phosphorylation of mitogen-activated protein kinase kinase (MKK)7 and c-Jun N-terminal kinase (JNK)-mediated apoptosis [21, 22]. Furthermore, RASSF7 overexpression in human nucleus pulposus cells decreases apoptosis [17], and RASSF7 expression is upregulated in tumors [14]. These observations suggest that RASSF7 is involved in the control of apoptosis, and has functions that are distinct from those of RASSF4, which inhibits lung cancer cell proliferation and invasion [16].

In the present study, we investigated the role of RASSF7 in non-small cell lung cancer (NSCLC) both *in vitro* and *in vivo*. We found that RASSF7 promotes NSCLC cell proliferation and migration by inhibiting Hippo signaling, which involves its coiled-coil domain.

## RESULTS

### Upregulation of RASSF7 expression in NSCLC correlates with poor prognosis

**Figure 1:**
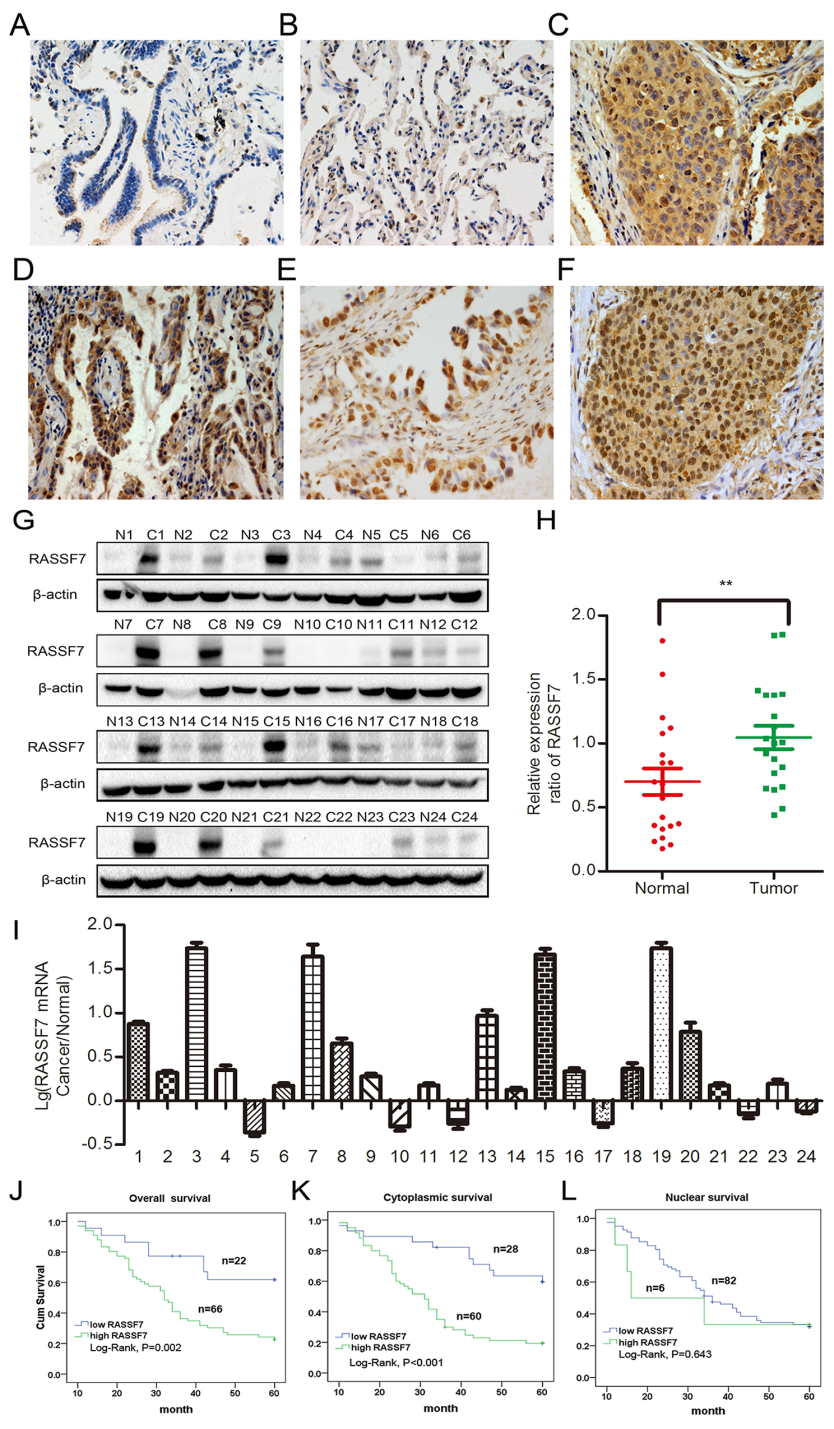
RASSF7 is highly expressed in NSCLC, which is correlated with poor prognosis RASSF7 presented negative or low expression in normal lung cancer **(A, B)**, but high cytoplasmic expression in lung squamous cell carcinoma **(C)** and adenocarcinoma **(D)**, which also showed positive RASSF7 nuclear expression **(E, F)** (400× magnification). Protein **(G, H)** and mRNA **(I)** levels of RASSF7 were higher in lung cancer than in paired adjacent tissue. Kaplan-Meier analysis demonstrated that patient overall survival negatively correlated with overall **(J)** and cytoplasmic **(K)**, but not nuclear **(L)** RASSF7 expression. β-actin was used as a loading control for western blotting. **P< 0.01 vs. control group (*t* test).

We performed immunohistochemistry to assess RASSF7 expression in 88 NSCLC and 20 paired noncancerous specimens. RASSF7 presented negative or low cytoplasmic expression in normal lung tissues (Figure 1A, 1B) and high cytoplasmic expression in carcinoma samples (Figure 1C, 1D), which also showed positive RASSF7 nuclear expression (Figure 1E, 1F). The frequencies of cytoplasmic and nuclear RASSF7 expression were 68.2% (60/88) and 6.8% (6/88), respectively, in NSCLC, and 20% (4/20) in normal lung tissues. Western blot of RASSF7 protein and quantitative (q)PCR of RASSF7 mRNA from 24 lung cancer tissue samples revealed that RASSF7 protein was overexpressed relative to paired adjacent tissues (0.97 ± 0.08 vs. 0.46 ± 0.06; *P* < 0.01) in 75% (18/24) of the samples (Figure 1G, 1H), with a similar percentage of samples showing upregulation of *RASSF7* mRNA (Figure 1I). We then analyzed the correlation between RASSF7 expression and prognosis. Statistical analyses revealed that overall RASSF7 and cytoplasmic RASSF7 expression correlated with high TNM stage (*P* = 0.001 and *P* < 0.001, respectively) and lymph node metastasis (*P* < 0.001 and *P* < 0.001, respectively). However, nuclear RASSF7 expression showed no obvious correlation with clinicopathological factors (Table 1). Kaplan-Meier analysis indicated reduced patient survival in tumors positive for overall and cytosolic RASSF7 expression (35.288±2.096 months and 33.258±2.059 months, respectively) as compared with RASSF7-negative patients (48.452±3.513 months, *P* = 0.002; 49.909 ± 3.133 months, *P* <0.001, respectively; Figure 1J, 1K). Survival of patients with and without nuclear RASSF7 was similar (32.833±8.355 months vs 38.955±1.939 months, *P* = 0.643; Figure 1L). Subsequent cox univariate (UA) and multivariate (MA) analyses revealed that cytoplasmic expression of RASSF7 could be considered an independent prognostic factor in NSCLC (*P* < 0.001 for UA, and *P* = 0.025 for MA, Table 2).

**Table 1:** Association between RASSF7 expression and clinicopathologic factors in NSCLC

Characteristics	N	Overall expression			Cytoplasmic expression			Nuclear expression		
		low	high	*P*	low	high	*P*	low	high	*P*
Age										
< 60 (years)	35	8	27	0.706	10	25	0.595	31	4	0.210
≥ 60 (years)	53	14	39		18	35		51	2	
Gender										
Male	34	10	24	0.448	9	25	0.393	31	3	0.673
Female	54	12	42		19	35		51	3	
Histology										
Squamous carcinoma	35	6	29	0.167	16	37	0.686	32	3	0.679
Adenocarcinoma	53	16	37		12	23		50	3	
Differentiation										
Well	30	9	21	0.436	9	21	0.792	29	1	0.659
Moderate/poor	58	13	45		19	39		53	5	
TNM stage										
I+ II	34	15	19	0.001	19	15	<0.001	34	0	0.078
III	54	7	47		9	45		48	6	
Lymph node metastasis										
Yes	19	11	8	<0.001*	13	6	<0.001	18	1	1.000
No	69	11	58		15	54		64	5	

RASSF7, Ras-association domain family 7; TNM, tumor-node-metastasis.

**Table 2:** Summary of Cox univariate and multivariate regression analysis of the association between clinicopathological features and overall survival in 88 cases of NSCLC

Characteristics	Hazard ratio (95% CI)	*p*
**Univariate analysis**		
Gender: male	1.036 (0.611-1.756)	0.896
Age older than 60	0.904(0.537-1.520)	0.703
Histological type: adenocarcinoma	0.924 (0.538-1.585)	0.773
Poor differentiation	1.414 (0.847-2.363)	0.186
High TNM classification	2.111 (1.198-3.718)	0.010
Positive lymph node metastasis	1.437 (0.745-2.770)	0.280
Nuclear expression	1.266(0.458-3.497)	0.649
Cytoplasmic expression	3.463 (1.782-6.730)	<0.001
Overall expression	3.002(1.421-6.342)	0.004
**Multivariate analysis**		
TNM classification	1.384 (0.764-2.509)	0.284
Cytoplasmic expression	2.408(1.117-5.189)	0.025
Overall expression	1.649(0.712-3.189)	0.243

NSCLC, non-small cell lung cancer.

### RASSF7 enhances NSCLC cell proliferation, migration and invasion

To investigate the function of RASSF7 in NSCLC, we evaluated the expression level of RASSF7 protein in a panel of lung cancer cell lines using western blot. In accordance with immunohistochemical results, RASSF7 protein expression was increased in 5/7 NSCLC cell lines (H1299, H292 Calu-1, LK2, and H661) compared with normal HBE cell line (Supplementary Figure 1).

**Figure 2:**
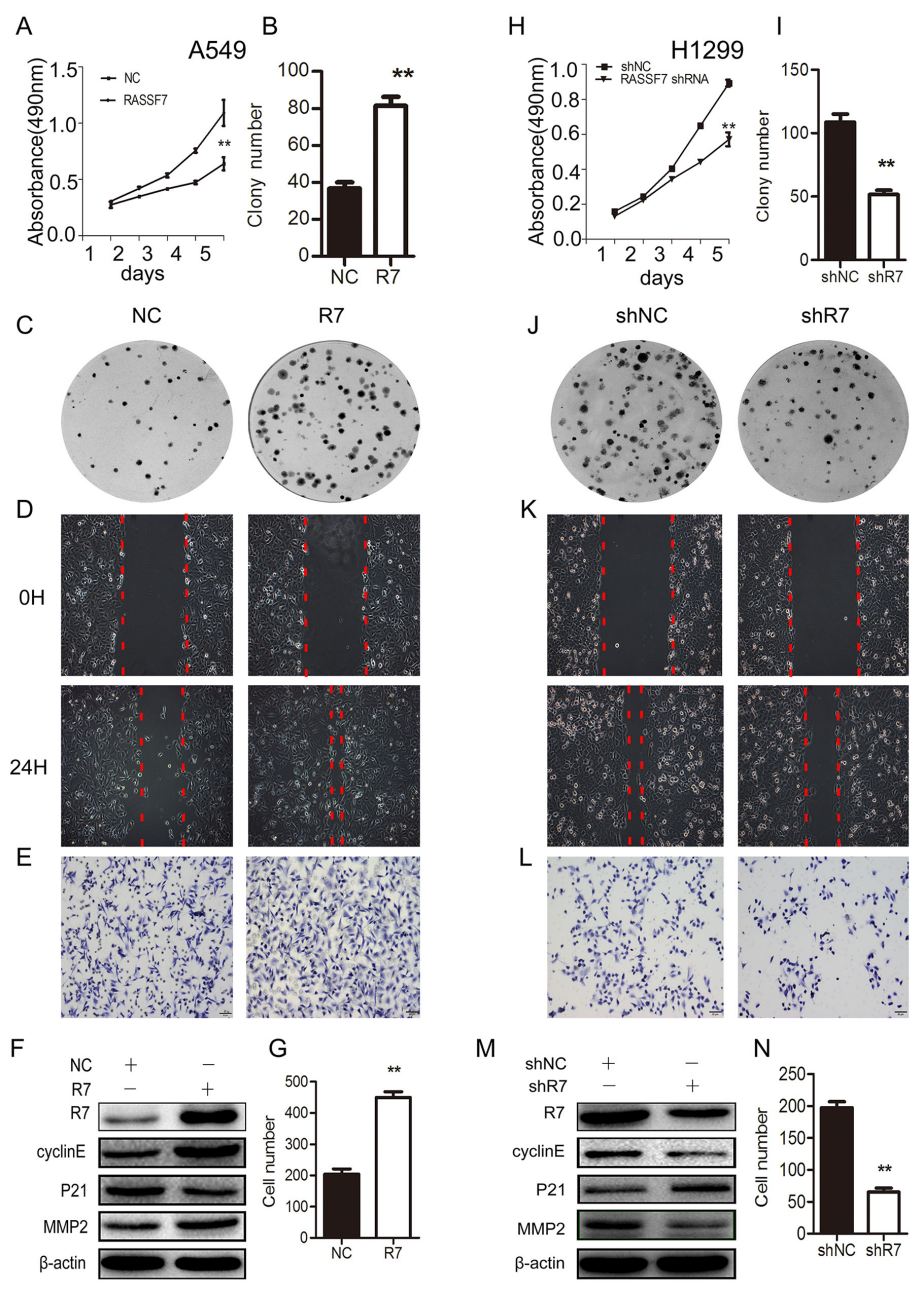
RASSF7 promotes NSCLC cell proliferation, migration, and invasion A549 cells were transfected with an RASSF7 overexpression plasmid and H1299 cells were transfected with RASSF7 (shR7) or a negative control (shNC) shRNA. Cell proliferation was evaluated with the MTT **(A, H)** and colony formation **(B, C, I, J)** assays. Cell migration was examined with the wound healing assay **(D, K)**. Cell invasion was evaluated with the matrigel invasion assay **(E, G, L, N)**. RASSF7, cyclin E, P21, and MMP2 protein expression was determined by western blotting **(F, M)**, with β-actin used as a loading control. **P< 0.01 vs. control group (*t* test).

We then transfected A549 cells (which have a low endogenous level of RASSF7) with a RASSF7 overexpression plasmid and knocked down RASSF7 expression in H1299 cells (which have a high endogenous level of RASSF7) with a RASSF7 short hairpin (sh)RNAshR7 (Supplementary Figure 2A). After checking RASSF7 overexpression and knockdown efficiency both in A549 and H1299 cells (Supplementary Figure 2C), we found that RASSF7 overexpression induced A549 cell proliferation, as determined by methylthiazolyldiphenyl tetrazolium (MTT) (Figure 2A) and colony formation assays [37 ± 3 vs. 81 ± 5 (control); *P* < 0.01] (Figure 2B, 2C). Cell migration (NC group: 0.35±0.03mm vs. R7 group: 0.82±0.02mm, *P* < 0.01) (Figure 2D) and invasion (Figure 2E, 2G) were also enhanced by RASSF7 overexpression, as determined by wound healing and matrigel invasion assays [204 ± 17 vs. 448 ± 19 (control); *P* < 0.01], respectively. Western blotting revealed that RASSF7 overexpression increased the levels of cyclin E, matrix metalloproteinase 2(MMP2) and decreased the levels of P21 (Figure 2F). On the other hand, RASSF7 shRNA transfection inhibited cell proliferation (109 ± 6 and 52 ± 3; *P* < 0.01) (Figure 2H–2J), migration (shNC group: 0.78±0.02mm vs. R7 group: 0.38±0.01mm, *P* < 0.01) (Figure 2K), and invasion relative to control cells (197 ± 10 vs. 65 ± 6; *P* < 0.01) (Figure 2L–2N), and reduced cyclin E, and MMP2 expression while increasing the levels of P21 (Figure 2M). Reversed results were obtained by transfecting A549 cells with RASS7 shRNA and H1299 cells with RASS7 plasmid (Supplementary Figure 3).

### RASSF7 increases NSCLC cell proliferation, migration and invasion via inhibition of Hippo signaling

**Figure 3:**
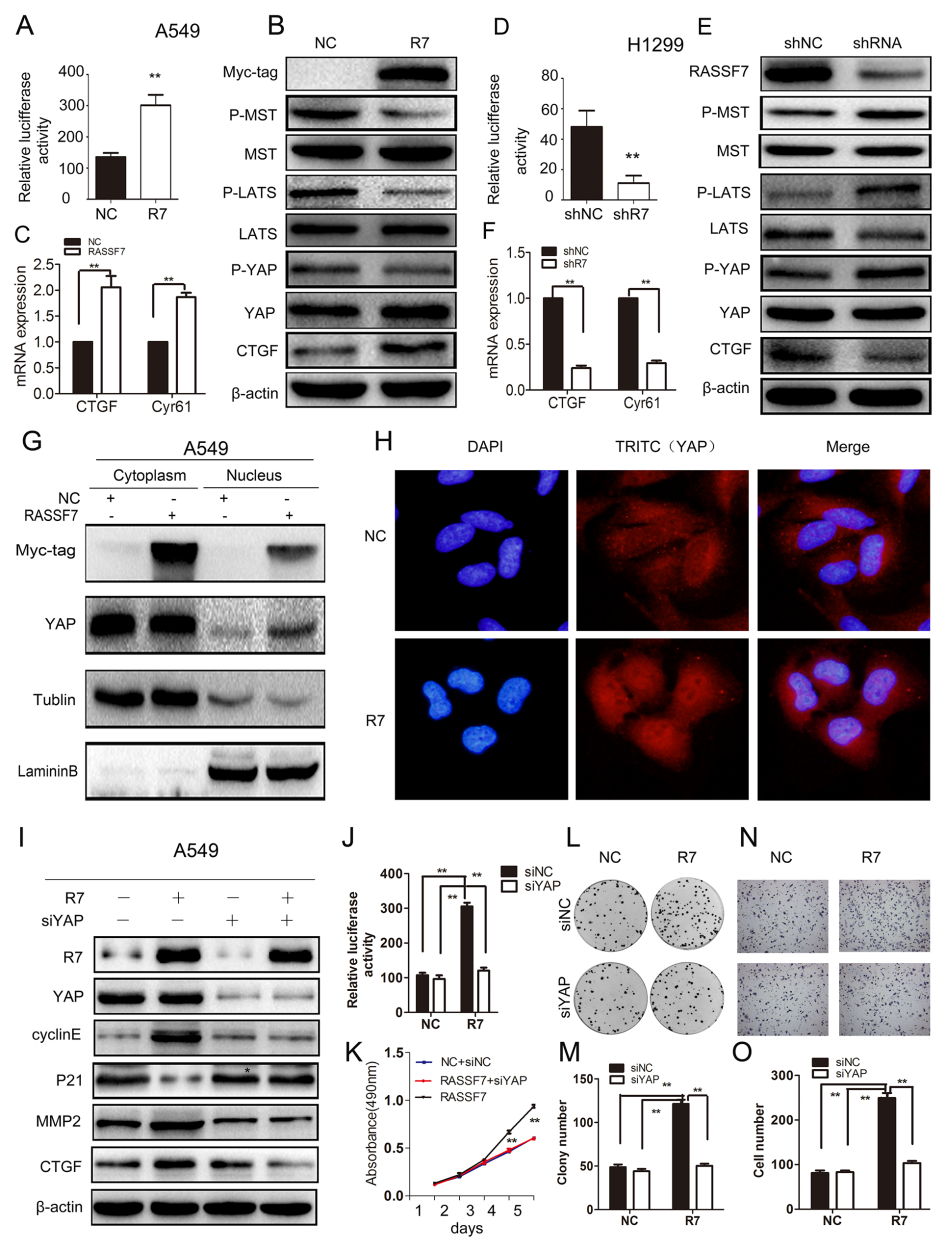
RASSF7 increases NSCLC cell proliferation, migration and invasion by inhibiting Hippo signaling **(A, D)** TEAD transcriptional activity was evaluated in A549 cells transfected with RASSF7 plasmid or H1299 cells transfected with RASSF7 shRNA with luciferase activity assay. **(B, E)** Phosphorylation of hippo signaling pathway components MST1/2, LATS1, and YAP and expression of the target protein CTGF were examined by western blotting. **(C, F)** *CTGF* and *Cyr61* mRNA levels were determined by qPCR. YAP protein nuclear translocation in A549 cells transfected with RASSF7 plasmid was evaluated by nuclear/cytoplasmic distribution and western blotting **(G)** and immunofluorescence analysis **(H)** (Magnification 400×). The effects of cotransfecting A549 cells with RASSF7 plasmid and YAP siRNA (siYAP-1) were examined by **(I)** western blotting, **(J)** luciferase activity assay (TEAD transcriptional activation), **(K)** MTT assay (proliferation), **(L, M)** colony formation assay (proliferation), and **(N, O)** matrigel invasion assay.

To investigate the mechanism by which RASSF7 promotes NSCLC cell proliferation, migration and invasion, we measured the transcriptional activity of the hippo signaling pathway transcription factor TEAD using a luciferase reporter assay. We also measured the protein levels of core hippo pathway components and mRNA levels of the hippo signaling targets connective tissue growth factor (CTGF) and cysteine-rich angiogenic inducer (Cyr) 61 in cells transfected with RASSF7 plasmid or RASSF7 shRNA. We found that RASSF7 overexpression enhanced the transcriptional activity of TEAD relative to control cells (135 ± 13 vs. 301 ± 34; *P* < 0.05) (Figure 3A), reduced the phosphorylation of MST1/2, LATS1, and YAP, and increased the expression of CTGF protein (Figure 3B) and *CTGF* and *Cyr61* mRNA (*P* < 0.01). The opposite trends were observed for TEAD transcriptional activity [46 ± 13 vs. 11 ± 5 (control), *P* < 0.05] (Figure 3D); MST1/2, LATS1, and YAP phosphorylation; CTGF protein expression (Figure 3E); and CTGF and Cyr61 mRNA levels (*P* < 0.01) (Figure 3F) upon RASSF7 knockdown. Western blot analyses of subcellular fractions revealed that RASSF7 overexpression increased the levels of YAP protein in the nucleus (Figure 3G), which was confirmed by immunofluorescence (Figure 3H).

To further test whether RASSF7 enhances NSCLC cell proliferation, migration and invasion via the hippo signaling pathway, we cotransfected A549 cells with RASSF7 plasmid and YAP-siRNA(siYAP-1) (Figure 3I–3O and Supplementary Figure 2B). We found that the expression of P21 increased whereas the expression of target genes CTGF, cyclin E, and MMP2 decreased (Figure 3I). Furthermore, the transcriptional activity of TEAD (Figure 3J), cell proliferation (Figure 3K–3M), and invasion (Figure 3N–3O) were inhibited. We also performed immunohistochemistry to assess the correlation between RASSF7 and p-YAP(s127) in 88 NSCLC tumor specimens (Supplementary Figure 4). The Spearman rank correlation test revealed that the expression of RASSF7 negatively correlated with the expression of p-YAP(s127) (correlation coefficient = −0.678, *P* < 0.001, Supplementary Table 1). In addition, we treated RASSF7-transfected cells with phosphatase inhibitor, okadaic acid (OA; 100 nM), and obtained the similar results as with YAP siRNA (Supplementary Figure 5). These results confirmed that RASSF7 promoted NSCLC cell malignant phenotype via inhibition of Hippo signaling.

### RASSF7 function is dependent on the CC domain

**Figure 4:**
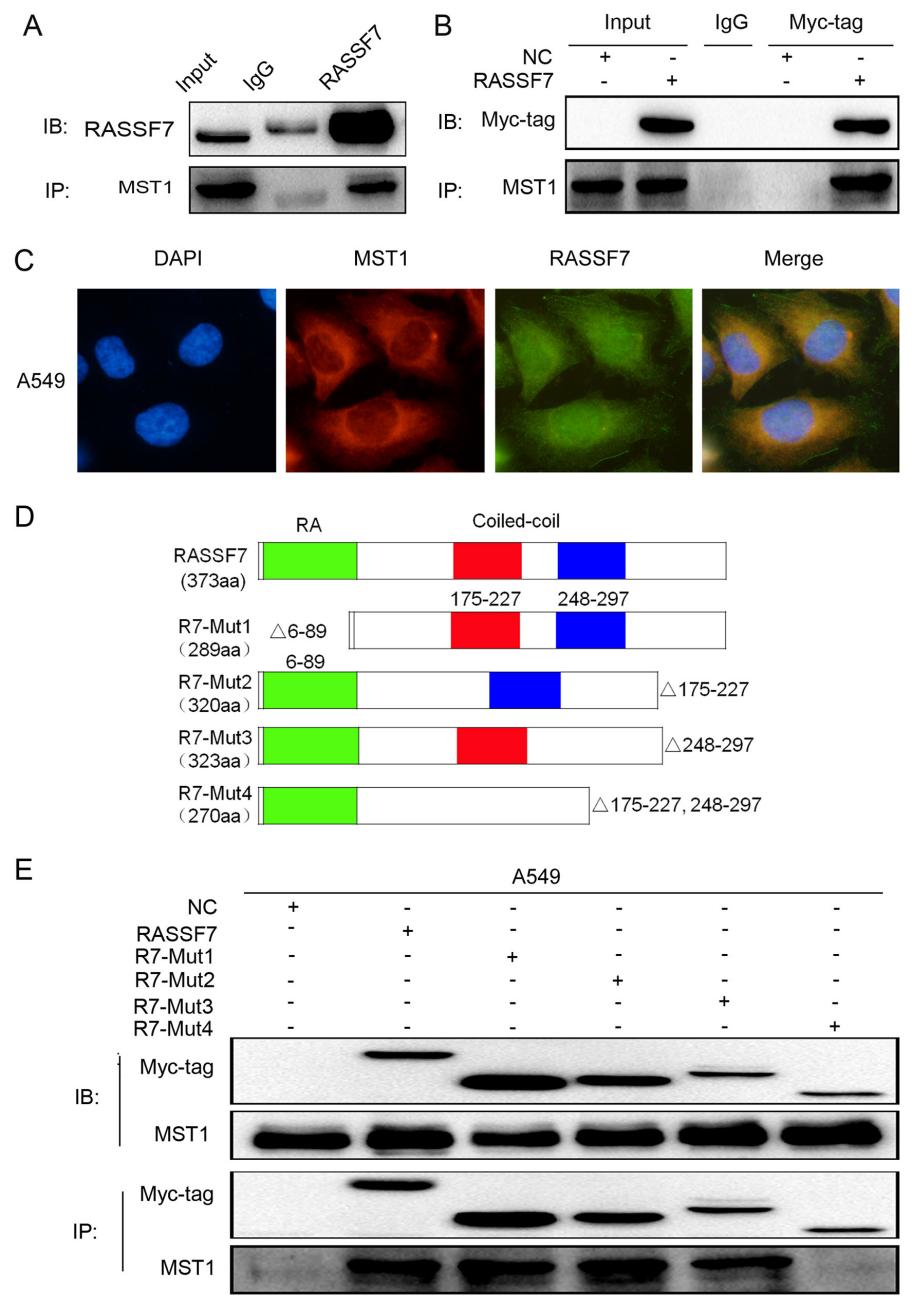
RASSF7 directly interacts with MST1 in A549 cells The interaction between endogenous RASSF7 and MST1 was analyzed by **(A)** co-immunoprecipitation and **(C)** immunofluorescence labeling(magnification 400×). **(B)** The interaction between MST1 and overexpressed RASSF7 was analyzed by co-immunoprecipitation. **(D)** Constructs for generating four RASSF7 mutants. **(E)** The interaction of mutant RASSF7 proteins with MST1 was examined by co-immunoprecipitation.

We confirmed that RASSF7 could interact with MST1 (Figure 4A, 4B) and that the two proteins co-localize in the cytoplasm (Figure 4C). We then transfected A549 cells with RASSF7 mutant constructs (Figure 4D) and found by co-immunoprecipitation that the interaction was abolished for the mutant protein lacking both RASSF7 CC domains (R7-Mut4) (Figure 4E).

**Figure 5:**
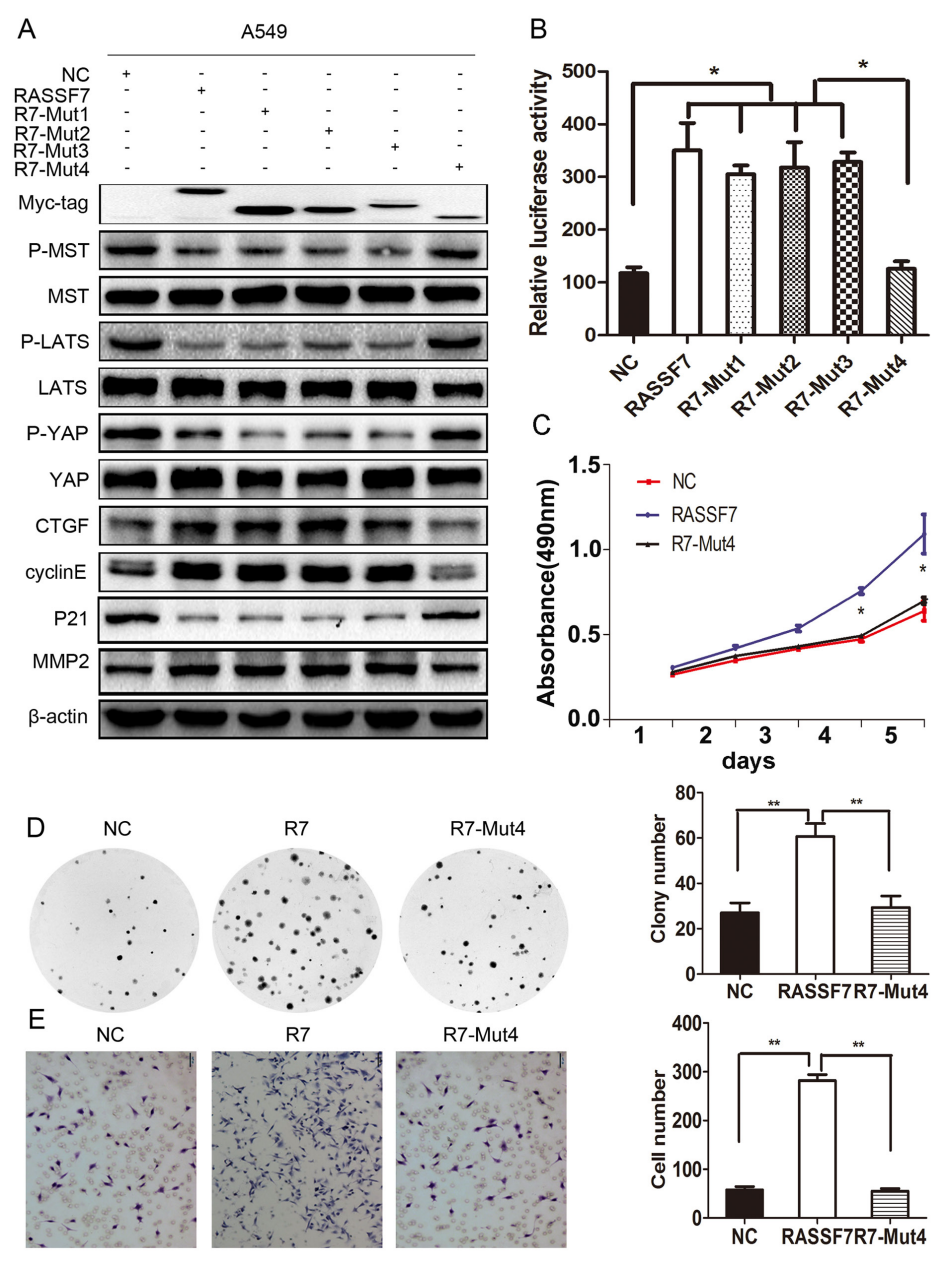
The biological function of RASSF7 depends on the CC domains **(A)** Western blot analysis of p-MST, p-LATS1, p-YAP, MST1, LATS1, YAP, CTGF, cyclin E, P21, and MMP2 levels. **(B)** The transcriptional activity of TEAD was evaluated with luciferase activity assay. **(C)** Cell proliferation was evaluated with the MTT and **(D)** colony formation assays. **(E)** Cell invasion was assessed with the Matrigel invasion assay. β-actin was used as a loading control. *P< 0.05, **P< 0.01 vs. control group (t test).

To further verify the results above, we evaluated the expression of core proteins of the Hippo pathway, TEAD transcriptional activity, and changes in cell proliferation, migration, and invasion. We found that the phosphorylation levels of MST1/2, LATS1, and YAP were restored only in cells expressing R7-Mut4 (Figure 5A). In addition, TEAD transcriptional activity was decreased (RASSF7: 21.95 ± 0.97, RASSF7-Mut1: 20.60 ± 0.40, RASSF7-Mut2: 22.29 ± 0.57, RASSF7-Mut3: 20.40 ± 1.02; RASSF7-Mut4: 11.07 ± 1.90; *P* < 0.05) (Figure 5B) while cell proliferation [61 ± 3 vs. 29 ± 3 (control), *P* < 0.01] (Figure 5C, 5D) and invasion [282 ± 12 vs. 55 ± 5 (control), *P* < 0.01] (Figure 5E) were inhibited upon transfection with the mutant constructs. These results confirmed that the CC domain was critical for the interaction of RASSF7 with MST1 and its promotion of NSCLC progression via Hippo signaling. Removal of the RA domain or a single CC domain had little effect on RASSF7 function.

### RASSF7 stimulates proliferation, migration and invasion of tumors transplanted into mice

**Figure 6:**
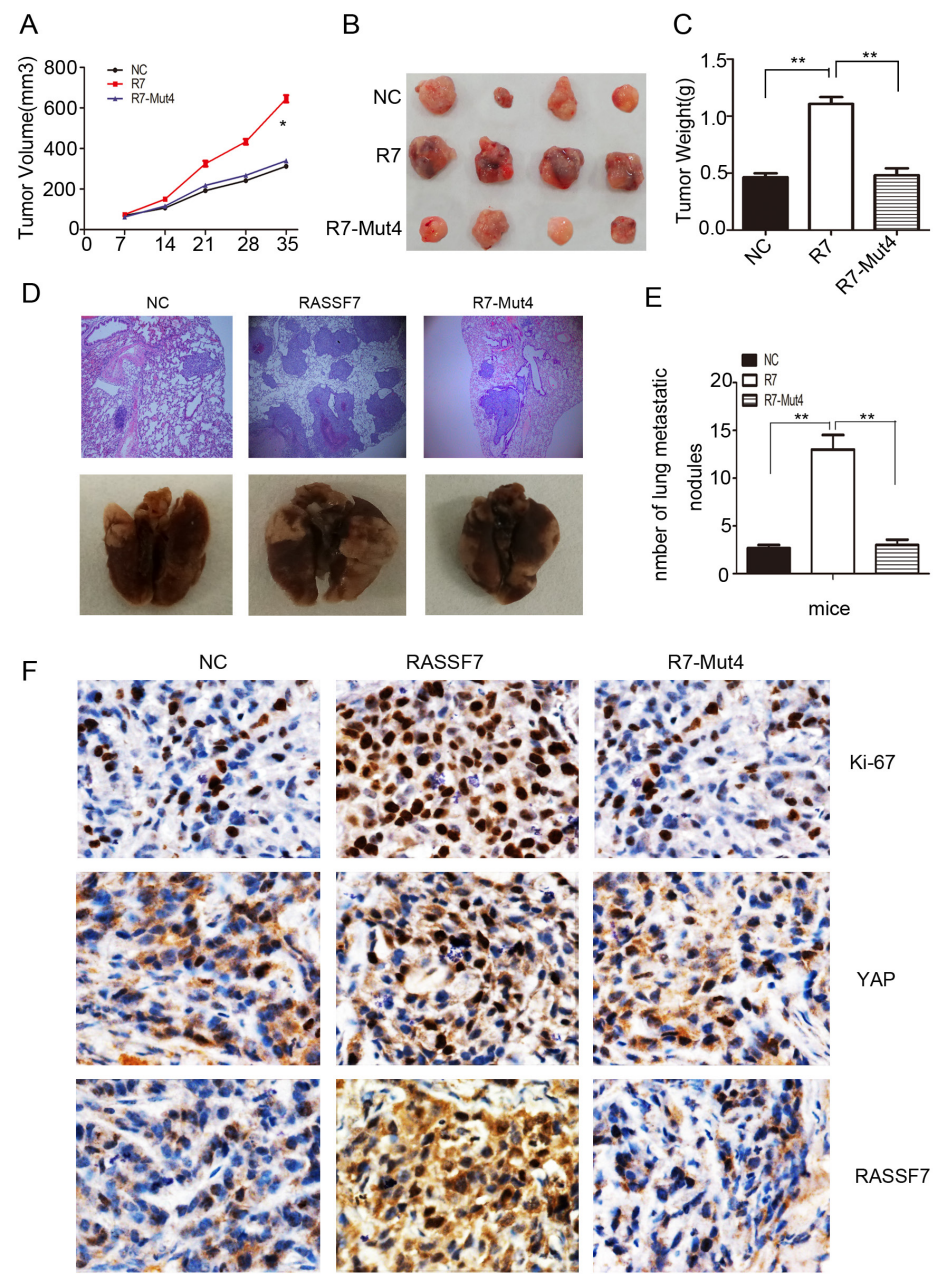
RASSF7 stimulates tumor proliferation and invasion in *vivo* Nude mice were injected with A549 cells transfected with a negative control vector (NC), full-length RASSF7, or RASSF7-Mut4 construct, and tumor growth was evaluated by measuring **(A)** volume and **(C)** weight. **(B, F)** Immunohistochemical analysis of Ki-67, YAP, and RASSF7 expression (magnification 400×). H&E staining was used to assess the invasion of cells transfected with **(D)** RASSF7 (magnification 100×) and **(E)** RASSF7-Mut4.

To evaluate the role of RASSF7 in NSCLC progression *in vivo*, nude mice were subcutaneously or intravenously injected with cells transfected with a negative control vector, full-length RASSF7, or RASSF7-Mut4 construct. Tumor volume (Figure 6A, 6B) and weight (Figure 6C), as well as the number of lung metastatic nodules (Figure 6D, 6E), were increased in mice transplanted with RASSF7-overexpressing cells relative to the other two groups. In contrast, the above metrics in the RASSF7-Mut4 group were comparable to the values observed in controls. To further validate the association between RASSF7 and Hippo signaling in NSCLC tissues, we examined the relationship between RASSF7 protein expression and Hippo component YAP, as well as Ki-67 expression in NSCLC specimens using immunohistochemistry. As shown in Figure 6F, both RASSF7 and Ki-67 staining of the controls and RASSF7-Mut4 group were weaker than those of the RASSF7-overexpressing group, and YAP localized mainly to the cytoplasm. On the other hand, both RASSF7 and Ki-67 staining became stronger and YAP localized to the nucleus in the RASSF7-overexpressing group.

## DISCUSSION

RASSF1A is the most widely studied of the RASSF proteins and is involved in cell cycle regulation and Ras-induced apoptosis [23]. The other classical RASSFs are often epigenetically inactivated, although RASSF6 is known to regulate the cell cycle and apoptosis [24]. Moreover, these proteins differ in terms of subcellular localization and mechanism of action. For example, RASSF1A and RASSF2 activate MST2 and induce apoptosis via Hippo signaling whereas RASSF6 inhibits MST1/2 and induces apoptosis through a different pathway, despite all RASSF proteins harboring an RA domain.

Although there are structural differences between classical and N-RASSFs, RASSF8 and RASSF10 also act as a tumor suppressors [25–27] while RASSF7 regulates cell growth and apoptosis [18]. Disrupted in schizophrenia 1 regulates astrogenesis in the embryonic brain via RASSF7 modulation of the RAS/MKK/extracellular signal-regulated kinase cascade [28]. RASSF7 is overexpressed in pancreatic and endometrial cancers and ovarian clear cell carcinoma [14]. However, the relationship between RASSF7 expression and clinicopathologic factors in human cancers has not been reported, and the precise role of RASSF7 in tumors is unknown. Our study here showed that RASSF7 presented low cytoplasmic expression in normal lung tissues and high expression in the cytoplasm of NSCLC. RASSF7 also presented high nuclear expression in lung cancer tissues, a finding that warrants further research. High cytoplasmic RASSF7 expression correlated with high TNM stage, lymph node metastasis, and poor patient prognosis. We also found that RASSF7 overexpression induced NSCLC cell proliferation, migration and invasion, which were inhibited by RASSF7 knockdown. These results indicate that RASSF7 functions as an oncogene.

Owing to their SARAH domain, classical RASSFs can combine with the Hippo pathway protein MST. However, this domain is absent in N-RASSFs [29–30]. Although the interaction between RASSF7 and MST1 has been previously demonstrated [13], the precise domain involved was not resolved. In the present study, we confirmed the interaction between RASSF7 and MST1 using co-immunoprecipitation and immunofluorescence. We found that RASSF7 and MST1 co-localize in the cytoplasm. Furthermore, among many RASSF7 mutants tested, only that lacking both CC domains (R7-Mut4) lost the ability to bind MST1. This restored p-MST1/2, p-YAP, p-LATS1, and P21 expression while reducing the levels of the downstream target genes CTGF and cyclin E, and inhibiting cell proliferation, migration, and invasion. Thus, RASSF7 interacts with MST1 through the CC domain to inhibit Hippo signaling, which does not require the RA domain.

RASSF7 overexpression increased TEAD transcriptional activity and concomitantly reduced the phosphorylation levels of MST1/2, LATS1, YAP, as well as P21 expression, and increased the expression of CTGF, cyclin E, and MMP2. We also found that nuclear translocation of YAP was increased. These effects were abolished by RASSF7 knockdown. When A549 cells were cotransfected with RASSF7 plasmid and YAP siRNA(siYAP-1) or transfected with RASSF7 plasmid in the presence of OA treatment, the phosphorylation levels of MST1/2 and LATS1, and the expression YAP all increased. The increase in cell proliferation, migration, and invasion induced by RASSF7 was also abrogated.

Our *in vivo* experiments demonstrated that tumor volume, weight, and the number of lung metastatic lesions were reduced in mice injected with R7-Mut4-transfected cells as compared to those transplanted with cells expressing full-length RASSF7. This corresponded to a decrease in the Ki-67 proliferation index and in YAP translocation.

Taken together, the results from our current study confirm that RASSF7 functions as an oncogene *in vivo* via inhibition of Hippo signaling, unlike classical RASSF1–6. These findings indicate that therapeutics that target RASSF7 might be effective in inhibiting NSCLC progression and could thereby improve patient prognosis.

## MATERIALS AND METHODS

### Information about patients, animals and specimens

All human specimens were obtained from patients who provided written, informed consent. The study protocol was approved by the ethics committee of China Medical University. Female BALB/C mice (four weeks old) were used in this study, and animal experiments were approved by the China Medical University Animal Care Committee.

A total of 108 tumor specimens including NSCLC tissue (n = 88) and paired non-tumor tissues (n = 20) (at a distance of > 5 cm from the edge of the primary tumor) were obtained at the First Affiliated Hospital of China Medical University between 2006 and 2008 by surgical resection. Patient survival was defined as the time from the day of surgery to the end of the follow-up period or the day of death due to recurrence or metastasis. Histological classification and lung cancer differentiation were evaluated according to 2015 World Health Organization classification criteria [30], and TNM staging of lung cancer was performed according to the 2009 Union for International Cancer Control standard. In addition, 24 fresh lung cancer tissue specimens (see Supplementary Table 2) were used to detect RASSF7 mRNA and protein levels. None of the patients had received chemo- or radiotherapy before tumor excision. Clinical data for patients are shown in Table 1.

### Immunohistochemistry

Tissue samples were fixed in 4% formalin, embedded in paraffin, and cut into 4-μm serial sections. Immunohistochemical staining was performed with the streptavidin-peroxidase method using a diaminobenzidene kit (MaiXin, Shenzhen, China). The following rabbit polyclonal primary antibodies were used: rabbit polyclonal rat anti-RASSF7 (Origene, 1:200 dilution), p-YAP Rabbit mAb (Cell signaling, 1:100 dilution), YAP Rabbit mAb (Cell signaling, 1:100 dilution), and rat anti-Ki-67 kit (Golden Bridge). Samples were incubated with the antibodies at 4 °C overnight. As a negative control, sections were incubated with phosphate-buffered saline (PBS) instead of the primary antibody.

RASSF7 expression was semi-quantitatively scored based on the percentage of expression and signal intensity. RASSF7 staining was located mainly in the cytoplasm of tumor cells. The intensity staining score was indicated as 0 (no staining), 1 (weak staining), or 2 (strong staining). Staining percentage was scored as 0 (0%), 1 (1–25%), 2 (26–50%), 3 (51–75%), and 4 (76–100%). Final scores were calculated as the multiplication of intensity staining times staining percentage and thus ranged from 0 to 8. RASSF7 status was regarded as low RASSF7 expression (score < 4) or high expression/overexpression (score ≥ 4). All tumor sections were randomly analyzed by two independent investigators. A total of 100 cells were counted in five randomly selected fields per section at 400× magnification.

### Hematoxylin and eosin (H&E) staining

Lung tissue specimens from nude mice were fixed with 4% paraformaldehyde and embedded in paraffin, and cut into 4-μm section that were deparaffinized in xylene and rehydrated in a graded series of alcohol. After H&E staining, the sections were dehydrated with alcohol, cleared with xylene, and visualized with light microscopy.

### Cell culture and transfection

HBE, A549, H1299, H292, H460, LK2, and H661 NSCLC cell lines were cultured with Roswell Park Memorial Institute 1640 medium (Gibco, Grand Island, NY, USA) containing 10% calf serum (Invitrogen, Carlsbad, CA, USA). Calu-1 cells were cultured in Mccoy’s 5A medium (Sigma, St. Louis, MO, USA) containing 10% calf serum at 37 °C and 5% CO_2_. The pCMV6 empty vector and pCMV6-RASSF7 plasmid were purchased from Origene; pCMV6-RASSF7-Mut, RASSF7 shRNA (RASSF7-homo-268, RASSF7-homo-1387, RASSF7-homo-731), and the negative control shRNA were purchased from Shanghai GenePharma (Shanghai, China); YAP siRNA was purchased from Guangzhou RIBOBIO(Guangzhou, China); pGL3b_8×GTIIC-luciferase and pRL-TK plasmids were from Addgene (Cambridge, MA, USA). Lipofectamine 3000 transfection reagent (Invitrogen) was used for cell transfections. Semi-stably transfected cell lines were screened for four weeks with G418.

### Colony formation assay

A total of 500 cells were seeded in a 6-cm dish 24 h after transfection and cultured for 14 days. Cells were washed with PBS, then fixed with ice-cold methanol and stained with hematoxylin. At least 50 cell colonies per dish were counted.

### Wound healing assay

Cells were seeded in 6-well plates at a density of 1 × 10^6^/well. Mitomycin C (20 μmol/l; Sigma) was added to each well followed by incubation for 2 h, and the cell layer was scratched with 100-μl micropipette tip. After rinsing the plates to remove free-floating cells and debris, culture medium was added followed by incubation for 24 h before relative closed distance was measured in three random microscope fields.

### Matrigel invasion assay

A 24-well Transwell chamber with a pore size of 8 μm was used (Dow Corning, Corning, NY, USA) with the upper chamber coated with Matrigel (BD Biosciences, Franklin Lakes, NJ, USA) and filled with serum-free medium. Cells were trypsinized 24 h after transfection and transferred to the upper Matrigel chamber in serum-free medium containing 1 × 10^5^ cells followed by incubation for 16 h. Non-invaded cells on the upper membrane surface were removed with a cotton tip, and cells that had passed through the filter were fixed with 4% paraformaldehyde and stained with hematoxylin. The number of invaded cells was counted in 10 randomly selected high-power microscopic fields.

### MTT assay

Cells were seeded in 96-well plates in medium containing 10% fetal bovine serum at about 1,000/well and cell viability was determined after 1, 2, 3, 4, and 5 d with the MTT assay. Briefly, 20 μl of 5 mg/ml MTT solution (Sigma) were added to each well for 4 h at 37 °C; the medium was then removed and 150 μl of dimethylsulfoxide was added to solubilize the resultant formazan crystals. The absorbance at 490 nm was measured on a spectrophotometer.

### Luciferase activity assay

To assess TEAD transcriptional activity, A549 or H1299 cells were seeded in 24-well plates, then transiently transfected with PGL3b-8× GTIIC, pRL-TK, or RASSF7, RASS7-Mut, or empty plasmid using Lipofectamine 3000. After 24 h, cells were lysed and luciferase activity was detected with the Dual-Luciferase reporter assay system (Promega, Madison, WI, USA). The activity of thymidine kinase Renilla served as an internal standard.

### RNA extraction and qPCR

Total RNA was extracted from lung tissue and cells with RNAi Plus (TaKaRa, Dalian, China) and reverse transcribed into cDNA; qPCR was carried out using the SYBR Premix Ex Taq II (Perfect Real Time) kit (TaKaRa, Dalian, China) on an H7900 Real-Time PCR system (Applied Biosystems) under the following conditions: 95 °C for 30 s, 95 °C for 5 s, and 60 °C and 30 s for 40 amplification cycles. Each sample was prepared in triplicate and the 2^−ΔΔCT^ method was used to quantitate *RASSF7* gene expression. The experiment was repeated three times.

### Western blotting

Western blotting was performed as previously described [16]. Nuclear/cytoplasmic protein were separated using NE-PER Nuclear and Cytoplasmic Extraction Reagents (Thermo scientific, USA) according to the manufacturer`s protocol. Antibodies against the following proteins were used for western blotting: RASSF7 (1/1000; sc-374431) and CTGF (1/500; sc-14939) (both from Santa Cruz Biotechnology, Santa Cruz, CA, USA); p-LATS (1/500; 8654S), p-MST1/2 (1/500; 3681S); p-YAP (S127) (1/500; 13008S); MST1 (1/500; 3682S); LATS1 (1/500; 3477S); YAP (1/500; 4912S); P21 (1/500; 2947T); MMP2 (1/500; 10373-2-AP);cyclin E (1/500; 3682S); β-actin (1/1000; 12262S)(all from Cell Signaling Technology, Danvers, MA, USA). Protein levels were calculated relative to those of β-actin, tubulin (1/500; AT819-1, Beyotime Institute of Biotechnology, Shanghai, China), or Lamin B (1/500; ab16048, Abcam, Cambridge, MA, USA). The mean values of experiments repeated three times were reported.

### Co-immunoprecipitation

The supernatant of cell lysates was collected by centrifugation. Antibody was added followed by rotation overnight at 4 °C. Protein A/G agarose beads (Beyotime Institute of Biotechnology) were added to the immune complex followed by rotation for 4 h at 4 °C. After centrifugation at 1000 rpm and 4 °C for 5 min, the supernatant was discarded and the precipitate was washed three times with ice-cold radioimmunoprecipitation assay buffer, resuspended in sample buffer, and boiled for 10 min to dissociate the immune complex from the beads. The supernatant was collected by centrifugation for western blotting.

### Immunofluorescence labeling

Cells were fixed with 4% paraformaldehyde for 15 min and incubated with 0.1% Triton X-100 for 15 min, followed by blocking with goat serum for 2 h at room temperature. The cells were then incubated overnight at 4 °C with antibodies against RASSF7 (1:100), MST1 (1:100), and YAP (1:100), followed by secondary antibody for 1 h at room temperature. Nuclei were counterstained with DAPI and samples were imaged with a confocal microscope (Olympus, Tokyo, Japan).

### Tumor xenograft

Female BALB/C mice (four weeks old) reared in a pathogen-free clean environment were injected subcutaneously (5 × 10^6^, 200 μl) or intravenously (2 × 10^6^, 100 μl) via the tail vein with A549 cells transfected with RASSF7, RASSF7-Mut4, or empty plasmid, after screening by G418, A549 cells (n = 4 mice per group). Tumor growth was evaluated every seven days, and tumor volume was calculated using the equation: length × width^2^/0.5. At 35 days post-injection, mice were euthanized and the tumors and lungs were excised, imaged, and tissue sections were obtained for H&E staining and immunohistochemistry.

### Statistical analysis

SPSS software version 16 was used for data analysis. Results are expressed as mean ± SD. The χ^2^ test was used to calculate the correlation between RASSF7 expression and clinicopathologic factors. The association between RASSF7 and p-YAP in the same specimen was analyzed using Spearman rank correlation test. The Kaplan–Meier method was used to estimate the probability of patient survival, and differences in the survival of patient subgroups were compared with Mantel’s log-rank test. The Cox regression model was used for multivariate analysis. Student’s *t*-test was used to compare other data. *P*<0.05 was considered to indicate statistical significance.

## SUPPLEMENTARY MATERIALS FIGURES AND TABLES



## Abbreviations

CC domain: coiled-coil domain
CTGF: connective tissue growth factor
Cyr61: cysteine-rich angiogenic inducer 61
H&E: hematoxylin and eosin
JNK: c-Jun N-terminal kinase
LATS1/2: large tumor suppressor kinase 1/2
MKK7: mitogen-activated protein kinase kinase 7
MMP2: matrix metalloproteinase 2
MST1: mammalian Ste20-like kinase 1
MTT: methylthiazolyldiphenyl tetrazolium
NSCLC: non-small cell lung cancer
OA: okadaic acid
RA domain: Ras domain
RASSF: Ras-association domain family
SARAH domain: Sav/RASSF/Hippo domain
Sav1: salvador family WW domain-containing protein 1
TAZ: transcriptional coactivator with a PDZ-binding domain
TEAD: TEA domain family member
TNM: tumor-node-metastasis
YAP: Yes-associated protein
