# Supplementary material for: The coiled-coil domain of oncogene RASSF 7 inhibits hippo signaling and promotes non-small cell lung cancer
Source: Oncotarget. 2017 Aug 12;8(45):78734–48. doi: 10.18632/oncotarget.20223 (PMC5667994; doi:10.18632/oncotarget.20223)
Supplement: Supplementary file 1 [file oncotarget-08-78734-s001.pdf]

The coiled-coil domain of oncogene RASSF 7 inhibits hippo signaling and promotes non-small cell lung cancer

SUPPLEMENTARY MATERIALS

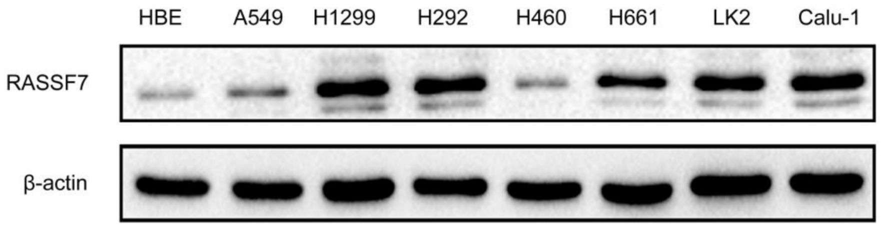

Supplementary Figure 1: Western blot analysis of RASSF7 protein expression in a panel of NSCLC cell lines. β-actin served as a loading control.

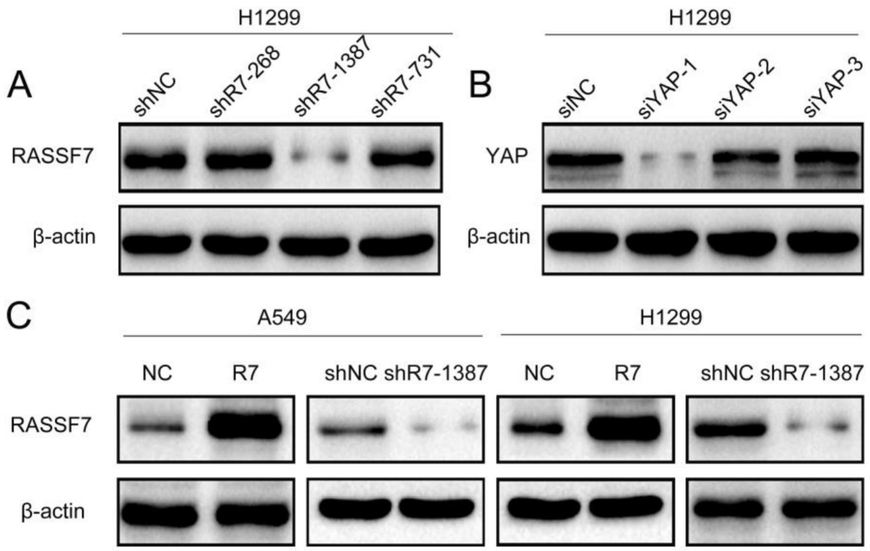

Supplementary Figure 2: RASSF7 overexpression and knockdown efficiency. **(A)** Three RASSF7 shRNA or **(B)** YAP siRNA sequences were used to knockdown RASSF7 or YAP in H1299 cells, respectively. We chose RASSF7-homo-1387 and siYAP-1 for subsequent experiments because they were the most efficient. **(C)** Western blot analysis showing RASSF7 overexpression and knockdown by shR7-1387 in A549 and H1299 cells.

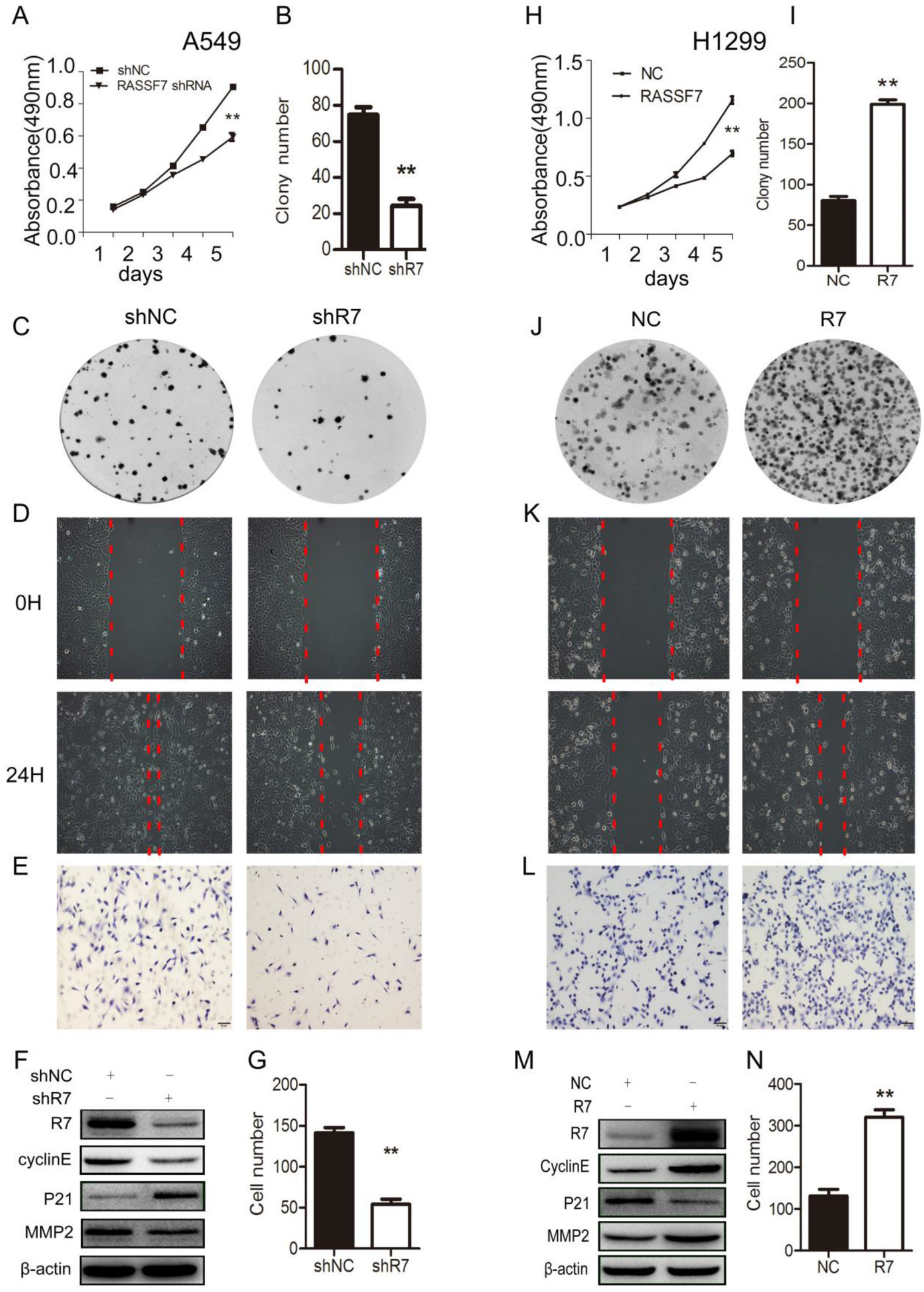

Supplementary Figure 3: RASSF7 promotes NSCLC cell proliferation, migration and invasion. A549 cells were transfected with RASSF7 (shR7) and H1299 cells were transfected with an RASSF7 overexpression plasmid. Cell proliferation was evaluated with the MTT **(A, H),** and colony formation **(B, C, I, J)** assays. Cell migration was examined with the wound healing assay **(D, K)**. Cell invasion was evaluated with the matrigel invasion assay **(E, G, L, N)**. RASSF7, cyclin E, P21, and MMP2 expression was measured using western blot **(F, M)**, with β-actin used as a loading control. **P < 0.01 vs. control group (*t* test).

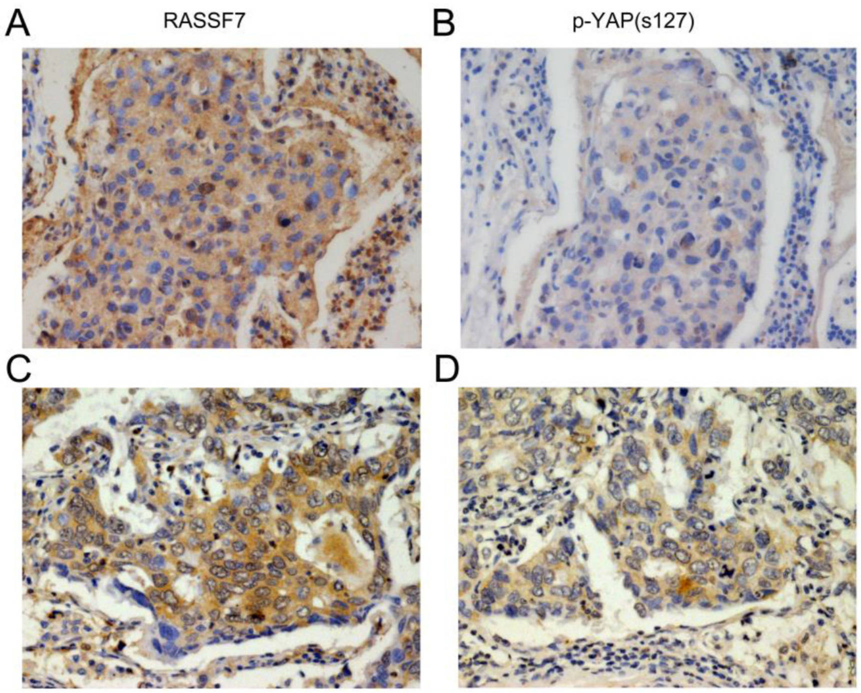

Supplementary Figure 4: Correlation between RASSF7 and p-YAP(s127) on the same specimens by immunohistochemistry. RASSF7 was highly expressed in SCC **(A)** and ADC **(C)** while p-YAP **(B, D)** presented low expression (magnification 400×).

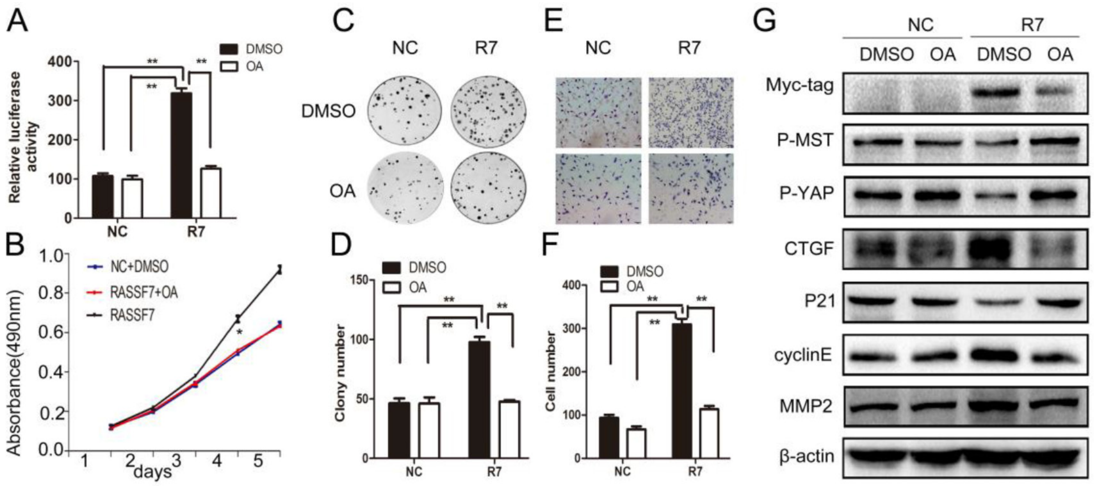

Supplementary Figure 5: OA treatment in A549 cells transfected with RASSF7 plasmid increases phosphorylation levels of MST and activates Hippo pathway activity. **(A)** Luciferase activity assay (TEAD transcriptional activation), **(B)** MTT assay (proliferation), **(C, D)** colony formation assay (proliferation), **(E, F)** matrigel invasion assay, and **(G)** western blot testing the effects of OA treatment in A549 cells transfected with RASSF7 plasmid.

Supplementary Table 1: Correlation between RASSF7 and p-YAP(s127) expression in NSCLC tissues

Characteristics	N	RASSF7 low expression	RASSF7 high expression	Correlation coefficient	*P*
p-YAP(s127)	88				
Low expression	72	4	68	-0.678	<0.001
High expression	16	11	5		

p-YAP(S127), phosphorylation of yes-associated protein(S127).

Supplementary Table 2: The clinical materials of 24 fresh lung cancer patients

Characteristics	Patients (n)	Positive expression of RASSF7
Age		
< 60 (years)	8	87.5%(7/8)
≥ 60 (years)	16	68.75(11/16)
Gender		
Male	15	73.3%(11/15)
Female	9	77.8%(7/9)
Histology		
Squamous carcinoma	10	70%(7/10)
Adenocarcinoma	14	78.6%(11/14)
Differentiation		
Well	15	66.7%(10/15)
Moderate/poor	9	88.9%(8/9)
TNM stage		
I+ II	16	75%(12/16)
III	8	75%(6/8)
Lymph node metastasis		
Yes	13	76.9%(10/13)
No	11	72.7%(8/11)
